# Testing the feasibility of a digital storytelling intervention combined with heart rate variability biofeedback in hematopoietic cell transplant patients

**DOI:** 10.1017/cts.2024.619

**Published:** 2025-01-22

**Authors:** Sunny W. Kim, Dara L. James, Rachel E. Koffer, Lakshmi Nair, Raheleh Bahrami, Lihong Ou, Veena Fauble, Nandita Khera, Linda K. Larkey

**Affiliations:** 1 Edson College of Nursing and Health Innovation, Arizona State University, Phoenix, AZ, USA; 2 College of Medicine, University of Michigan, Ann Arbor, MI, USA; 3 Hematology Oncology/Blood and Marrow Transplant, Mayo Clinic Arizona, Phoenix, AZ, USA

**Keywords:** Heart rate variability, heart rate variability biofeedback, digital storytelling, psycho-emotional well-being, stem cell transplant patients, distress

## Abstract

This pilot 2-week, randomized controlled trial examined integrating digital storytelling (DST) with heart rate variability biofeedback (HRVB) to enhance psycho-emotional well-being of hematopoietic cell transplantation (HCT) patients. HCT patients (*N* = 25; *M*_age_ = 51.9 years) were randomly assigned: (1) DST + HRVB intervention, or (2) DST-only control. Both groups viewed four emotionally-rich digital stories. The DST + HRVB group practiced HRVB at home for ten minutes/day. DST + HRVB participants decreased anger, depression, fatigue (*d* = 0.53) and increased emotional processing (*d* = 0.20), and HRV-assessed autonomic nervous system balance (3.5 vs. 0.9). This study supports feasibility of integrating DST with HRVB, and effect sizes indicate superiority of combining DST with HRVB.

## Introduction

Hematopoietic cell transplantation (HCT) treats hematologic and nonhematologic malignancies, such as lymphoma and certain solid tumors [1]. In 2020, 22,013 HCTs were performed in the USA [1]. Recipients surviving ≥ 2 years have an 80% 10-year survival rate [2]. However, HCT is aggressive, leading to significant distress; approximately 50% of patients experience long-term issues including depression and anxiety [3]. Effective interventions that address these concerns are essential [4], but identifying interventions that fit the medical restrictions and time/transportation constraints of HCT patients is challenging. Accessible and time efficient technology-based interventions are needed.

Storytelling interventions based on Narrative Theory have shown promise for alleviating distress in cancer and HCT patients [5,6]. Digital storytelling (DST) involves creating first-person audio-visual narratives of a clinically challenging experience that synthesize images, audio, music, and text [7]. Viewing these narratives in a remotely delivered intervention can reduce psychological distress by fostering emotional engagement and identification with the story [8] and can enhance emotional health by promoting emotional processing [9]. DST interventions can help patients accept and adjust to their emotions, potentially improving mood and reducing psycho-emotional distress.

Recent studies have used another accessible intervention that can be remotely delivered, heart rate variability biofeedback (HRVB) to improve psychological stress, cardiovascular resilience, and longevity [10]. Greater variability in the time intervals between heart beats and increased shifts to a rhythm called “coherence” indicate greater balance in the autonomic nervous system (ANS) and improved emotional well-being and resilience [10] [11] [12]. HRVB involves slowed-down breathing, a focus on positive emotions, and using visual and/or auditory feedback to “learn” to optimize these HRV patterns and the accompanying emotional and neurophysiological responses [13].

Combined with DST, HRVB can enhance cognitive appraisal, emotional processing, and emotional regulation. We hypothesized that HCT patients in an integrated DST + HRVB intervention would show greater improvements in psycho-emotional well-being, emotional processing, and ANS balance than those in a DST-only control condition at post-intervention.

## Methods

### Overview

This study used a double-blinded, randomized controlled trial (RCT) to examine the feasibility and preliminary effects of a 2-week DST enhanced with HRVB intervention (DST + HRVB) compared to a DST-only control condition in HCT patients (Clinicaltrials.gov ID: NCT04275830). Data were collected online using REDCap at two time points: 1) baseline (T1) and 2) post-intervention (T2) (two weeks following baseline). Participants were recruited between January 2020 and November 2021, and the study was approved by the IRB at both a cancer center and partnering university in the Phoenix Metropolitan area.

### Recruitment, eligibility, and consent

Adult HCT patients (18 years or older) within three months post-HCT discharge were invited to participate. Exclusion criteria included inability to comply with study protocol, visual or hearing impairments, conditions interfering with accurate HRV data detection, and regular practice of meditative/breathing exercises. Participants were recruited to the study without knowing that one arm was expected to improve emotional outcomes. Potential participants were identified through medical records and invited during clinic visits. Initial screening was performed based on the eligibility criteria. Those who agreed and eligible were screened further and provided informed consent in person.

### Study procedures

After consent, participants completed online surveys on psycho-emotional well-being in the clinic. HRV assessments were conducted using ear sensors and the emWave Pro Plus device, both at baseline and post-intervention. Participants were randomly assigned to either the DST + HRVB intervention group or the DST-only control condition with an HRVB waitlist.

### Data collection and outcome measurement

Each participant received weekly emails with REDCap links to modules containing two stories and short questionnaires. Participants were provided a $20 gift card. Data collectors and the statistician were blinded to study arm assignment.

#### Demographics

Demographic characteristics were assessed, including age, gender, ethnicity, income, education, transplant type, cancer diagnosis, health insurance, marital status, employment status, and support system.

**Psycho-emotional well-being** was operationalized as mood and emotion processing. *Mood* was measured using the Profile of Mood States (POMS)-short form (15 items) [14]. Respondents rated each item from 0 (not at all) to 4 (extremely), with a Cronbach’s alpha of 0.93 [9].

**Emotional Processing** was measured using the emotional approach coping scale, rated on a four-point response options (1 = I usually do not do this at all; 4 = I usually do this a lot), with a Cronbach’s alpha of 0.72 to 0.82 [9].

#### HRV parameters

HRV data were collected using the emWave Pro Plus device with a 3-minute “neutral” resting protocol. The Inner Balance app tracked home practice, with data retrieved from Heart Cloud accounts. HRV parameters, including standard deviation of normal to normal (SDNN), root mean square of successive differences (RMSSD), low frequency (LF), high frequency (HF), and normalized coherence, were collected at baseline and post-intervention.

### Description of the interventions

#### DST intervention

Both groups received weekly emails with links to a web-based DST intervention. Over two weeks, participants watched two DST videos per week (total 4 videos). These digital stories, developed in previous studies, were 3–5 minutes long and covered themes like transplant and recovery, coping with pain, spiritual support, and family support [6,15]. Participants in the DST-only group received HRVB training and modules after T2.

#### DST + HRVB intervention

Participants in the DST + HRVB group received the same DST videos and weekly reminder. They also received an Inner Balance HRV sensor, a smartphone app, and a 30-minute HRVB training session on generating a resonance frequency (RF) pattern of HRV, standardized by the HeartMath Institute (HMI)®. Participants were given a manual and instructed to practice HRVB skills daily for 10 minutes at home.

### Data analysis

Feasibility was measured by recruitment and retention rates, proposing benchmarks of 50% recruitment goals and 70% retention goals to be met. HRVB compliance was also measured as part of the feasibility. Descriptive statistics summarized sample characteristics. Changes from baseline to post-intervention were examined using multivariate linear regression, predicting the change score (Δ) by group (DST + HRVB versus DST) and adjusting for T1 score. Due to a relatively small sample size, age and gender were used as covariates. Here we focus our results on estimates of standardized between-group differences (Cohen’s d) in Δs when describing intervention effects on outcomes. Analyses were conducted in SPSS version 27 and R version 3.5.2.

## Results

### Sample description

Participants’ sociodemographic and other background characteristics are summarized in Table 1. Participants (*N* = 25, mean age = 51.9 years) were 10 female and 15 male adult cancer patients who had recently undergone HCT. The majority were White (76%), married (64%), and unemployed (52%). Most participants had undergone autologous transplantation (72%). The intervention group did not differ from those in the control group with respect to demographic characteristics.

**Table 1.** Demographic characteristics of the sample (N = 25)

		Frequency	
Variable	Total (n = 25)	Intervention Group (n = 13)	Control Group (n = 12)
			
Age (mean ± SD)		49.54 ± 16.74	54.58 ± 13.74
Gender			
Female	10	4 (30.8)	6 (50.0)
Male	15	9 (69.2)	6 (50.0)
Education level			
High School	3	2 (15.4)	1 (8.3)
Some College	10	6 (46.2)	4 (33.3)
4-year College	7	2 (15.4)	5 (41.7)
Master’s degree	5	3 (23.1)	2 (16.7)
Marital status			
Married	16	8 (61.5)	8 (66.7)
Never married	3	3 (23.1)	–
Divorced, widowed or separated	4	1 (7.7)	3 (25.0)
Not married/in a committed relationship	2	1 (7.7)	1 (8.3)
Employment status			
Employed full time	12	7 (53.8)	5 (41.7)
Not employed outside the home	13	6 (46.2)	7 (58.3)
Employment status change			
No	17	9 (69.2)	8 (72.7)
Yes	7	4 (30.8)	3 (27.3)
Type of health insurance			
Uninsured	1	1 (7.7)	–
Private coverage	13	8 (61.5)	5 (41.7)
Medicaid	1	–	1 (8.3)
Medicare	9	3 (23.1)	6 (50.0)
Other	1	1 (7.7)	–
Ethnicity			
Non-Hispanic/ Latino	17	12 (92.3)	5 (41.7)
Hispanic/ Latino	6	1 (7.7)	5 (41.7)
Race			
White	19	11 (84.6)	8 (66.7)
Asian/ Pacific Islander	1	1 (7.7)	–
Hispanic/Latino	5	3 (7.7)	4 (33.3)
Type of Transplant			
Autologous	18	10 (76.9)	8 (66.7)
Allogeneic	7	3 (23.1)	4 (33.3)
Cancer more than once			
No	18	11 (84.6)	7 (63.6)
Yes	6	2 (15.4)	4 (36.4)
Relation in social network			
Spouse	16	9 (69.2)	7 (58.3)
Sibling	6	3 (23.1)	3 (25.0)
Son	3	2 (15.4)	1 (8.3)
Daughter	4	2 (15.4)	2 (16.7)
Friend	7	4 (30.8)	3 (25.0)
Parents	5	3 (23.1)	2 (16.7)
Other	2	1 (7.7)	1 (8.3)
Support provided			
Assistance with symptom management	12	8 (61.5)	4 (33.3)
Assistance with decision making	11	7 (53.8)	4 (33.3)
Emotional support	18	12 (92.3)	6 (50.0)
Financial support	5	3 (23.1)	2 (16.7)
Spiritual support	8	3 (23.1)	5 (41.7)
Assistance with personal care	10	7 (53.8)	3 (25.0)
Practical support	15	9 (69.2)	6 (50.0)
Physical support	16	10 (76.9)	6 (50.0)
Assistance with child rearing/ parenting	4	2 (15.4)	2 (16.7)

### Feasibility of recruitment

**Figure 1.**
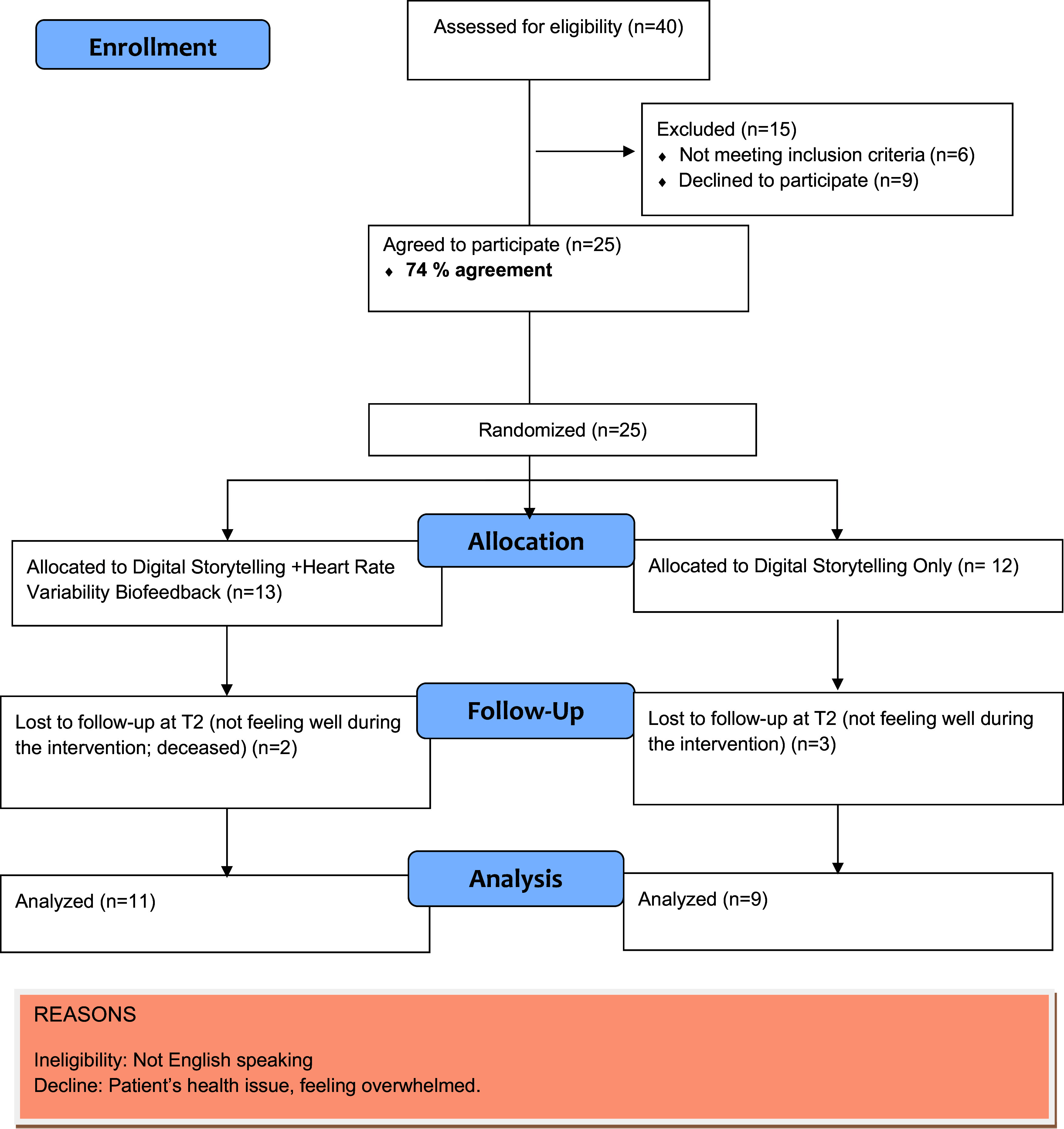
Enrollment flow consort diagram.

Figure 1 illustrates study flow and addresses feasibility of recruitment and retention benchmarks. Among patients approached and reached, (*N* = 40), nine patients declined to take part because of not feeling well and six were ineligible due to non-English speaking. Of 34 patients (74%), 25 agreed to participate; 13 patients were randomized to the intervention group, and 12 patients were randomized into the control group. Of the 25 patients who enrolled in the study, 5 did not reach T2. Retention was high: 20 (87%) completed the T2 assessment. For our feasibility benchmarks, all recruitment, retention, and data completion rates exceeded our goal of 50% recruitment and 70% of retention. Participants in the integrated DST + HRVB intervention group completed an average of 106 minutes of HRVB practice (mean = 7.57 min per day), indicating compliance.

### Effects of DST enhanced with HRVB

#### DST + HRVB improved psycho-emotional well-being

The integrated DST intervention participants showed improvement (medium to large effect sizes) in reduced anger (*d* = 0.61), depression (*d* = 0.44), and fatigue (*d* = 0.30) and a slight increase (small effect size) in emotional processing (*d* = 0.20), relative to the DST-only control group. Intervention effects on change in scores were small to moderate (0.20 ≤ *ds* ≤ .61). Specifically, ratings of POMS fatigue decreased for the DST + HRVB intervention group (Δ = −0.42) but increased for the DST-only control group (Δ = 0.78). Depression and anger decreased from pre- to post-intervention for both groups. The average decrease in depression and anger scores for the DST + HRVB intervention group (Δ = −0.88, Δ = −0.89, respectively) was significantly greater than that for the DST-only control group (Δ = −0.33, Δ = −0.35, respectively). Table 2 summarizes results of the standardized mean differences (ds) of baseline-adjusted outcome change scores.

**Table 2.** Unadjusted means and standard deviations for outcomes by group (DST + HRVB vs. DST) and time point (T1 vs. T2), with standardized mean differences (ds) of baseline-adjusted outcome change scores

	DST + HRVB (n = 11)		DST only (n = 9)		
Outcome measures	Pretest (M, SD)	Posttest (M, SD)	Pretest (M, SD)	Posttest (M, SD)	*d*
Emotional processing	3.14 (0.81)	3.38 (0.75)	3.01 (0.42)	3.13 (0.74)	0.20
POMS anger	1.33 (0.88)	0.44 (1.15)	1.20 (1.18)	0.85 (0.90)	0.61
POMS depression	1.73 (1.45)	0.85 (1.38)	1.16 (1.33)	0.83 (1.13)	0.44
**POMS fatigue**	**2.20 (1.11)**	**1.78 (1.37)**	**1.83 (0.80)**	**2.61 (1.50)**	**0.30**

*Note:* DST = digital storytelling; HRVB = heart rate variability biofeedback; *M* = mean; SD = standard deviation; *d* = effect size (Cohen’s *d*); POMS = profile of mood states.

#### DST + HRVB improved HRV

The DST + HRVB intervention group yielded notable differences (medium effect size) in HRV-assessed ANS balance (assessed using HRV normalized coherence; *d* = .55) compared to the DST-only control group with the integrated group increasing by 3.5 from pre- to post-intervention, while the DST-only control group increased by only 0.9. Ratings of time domain of HRV parameters (SDNN *d* = 0.39 and RMSSD *d* = .50) increased for the DST + HRVB intervention group but decreased for the DST-only control group. Full results of HRV Parameters are presented in Table 3.

**Table 3.** Group comparisons on HRV parameters

	Intervention Group (n = 11)		Control Group (n = 9)		
Outcome measures	Baseline (M, SD)	Post (M, SD)	Baseline (M, SD)	Post (M, SD)	d
SDNN	84.66 (93.26)	117.46 (77.80)	99.16 (78.49)	92.15 (61.48)	0.39
RMSSD	102.16 (114.05)	152.30 (112.76)	118.73 (115.71)	117.78 (79.10)	0.50
LF	1868.58 (4231.23)	2961.42 (3766.01)	1625.61 (2895.38)	2578.76 (3071.22)	0.14
HF	1433.56 (3461.14)	1767.94 (2049.58)	716.60 (1219.83)	1081.76 (1607.05)	0.15
NC	30.68 (7.35)	34.23 (4.89)	31.40 (9.76)	31.49 (7.54)	0.55

*Note: M* = mean; SD = standard deviation; *d* = effect size (Cohen’s *d*); SDNN = standard deviation of normal to-normal; RMSSD = root mean square of successive differences; LF = low frequency; HF = high frequency; NC = normalized coherence.

## Discussion

This pilot RCT examined the feasibility and preliminary effectiveness of an integrated DST + HRVB intervention to improve psychological distress and emotional processing in patients following HCT, compared to a DST-only control group. Given that all recruitment, retention, and data completion rates exceeded our goal, our findings suggest that the DST + HRVB intervention is feasible. Participants in the integrated DST + HRVB group reported great improvements in reduced anger, depression, fatigue, and increased emotional processing than those in the DST-only control group. This aligns with literature suggesting that focusing on improving psycho-emotional well-being and how patients undergoing aggressive cancer treatments can cope with treatment-related distress through HRV coherence and DST intervention videos [13,16]. HRVB may serve as a mindfulness approach, improving emotion regulation by calling attention to and accepting emotions felt during DST [12,17].

While the specific mechanism by which HRVB may enhance a psychological intervention was not tested, the study adds evidence that incorporating a neurophysiological component can strengthen the effects of reducing depression [18]. Additionally, the study identified a vulnerable patient population that may benefit from this dual approach. The complementary nature of DST and HRVB in addressing emotional processing likely contributes to the improvements. Personal narratives in DST fosters empathy and connection, while HRVB enhances emotional regulation and ANS balance, allowing patients to better manage stressors during HCT treatment. The increase in ANS balance in the DST + HRVB group suggests that HRVB may have facilitated a shift toward adaptive stress responses, reducing psycho-emotional distress and improving emotional well-being These findings are consistent with literature on the role of HRV in emotion regulation [13]. Further research should include more positive components of well-being to elucidate the specific benefits of HRVB. The pilot results support the potential of the DST + HRVB intervention in addressing psycho-emotional distress in HCT patients, targeting both psychological and physiological aspects of well-being.

This digital delivery of this intervention reduces barriers to attending in-person or scheduled online classes, making it particularly beneficial for HCT patients who face infection prevention challenges [19]. Technology-based online interventions are increasingly used to improve psycho-emotional well-being, offering flexibility in timing and location and widespread internet use [19]. This remotely delivered intervention is affordable, has minimal risk of side effects, and is especially beneficial for vulnerable patient populations [17]. Future studies should assess potential effects on caregiver coping, given the elevated levels of distress and burden reported among caregivers of HCT patients [5]. However, the pilot RCT has noted limitations, such as a small sample size, limiting generalizability, and was conducted in one geographical location with individuals who could afford appropriate HCT care. A longer intervention may yield more robust changes, and additional physical and psycho-emotional measures would be beneficial.

## Conclusions

Despite these limitations, this pilot RCT contributes meaningfully to the growing literature on psycho-emotional interventions for HCT patients. It demonstrates the feasibility of recruitment and retention, and the preliminary effectiveness of integrated DST + HRVB as a psycho-emotional intervention. Future research with larger sample sizes and longer follow-up is needed to confirm these findings and underlying mechanisms. This study addresses key research gaps and provides evidence to inform the development and testing of psycho-emotional interventions for patients with other cancers and diseases. Future work should extend the current study for broader dissemination of this potentially distress-relieving intervention via a low-cost, flexible, noninvasive, and portable approach to improve psycho-emotional well-being.
